# Effects of Different Stress Parameters on Growth and on Oleuropein-Degrading Abilities of *Lactiplantibacillus plantarum* Strains Selected as Tailored Starter Cultures for Naturally Table Olives

**DOI:** 10.3390/microorganisms8101607

**Published:** 2020-10-19

**Authors:** Amanda Vaccalluzzo, Alessandra Pino, Maria De Angelis, Joaquín Bautista-Gallego, Flora Valeria Romeo, Paola Foti, Cinzia Caggia, Cinzia L Randazzo

**Affiliations:** 1 Department of Agricultural, Food and Environment, University of Catania, 95123 Catania, Italy; amanda.vaccalluzzo@unict.it (A.V.); paola.foti@phd.unict.it (P.F.); ccaggia@unict.it (C.C.); 2 Department of Soil, Plant and Food Science, University of Bari Aldo Moro, 70121 Bari, Italy; maria.deangelis@uniba.it; 3 Faculty of Science—University of Extremadura, 06006 Badajoz, Spain; joaquinbg@unex.es; 4 Research Centre for Olive, Fruit and Citrus Crops (CREA), 95024 Acireale, Italy; floravaleria.romeo@crea.gov.it

**Keywords:** table olives fermentation, growth ability, beta-glucosidase, oleuropeinolytic activity, low salt content

## Abstract

The use of β-glucosidase positive strains, as tailored-starter cultures for table olives fermentation, is a useful biotechnological tool applied to accelerate the debittering process. Nowadays, strains belonging to *Lactiplantibacillus plantarum* species are selected for their high versatility and tolerance to stress conditions. The present study investigated the effect of different stress factors (pH, temperature and NaCl) on growth and on oleuropein-degrading abilities of selected *L. plantarum* strains. In addition, the presence of the beta-glucosidase gene was investigated by applying a PCR based approach. Results revealed that, overall, the performances of the tested strains appeared to be robust toward the different stressors. However, the temperature of 16 °C significantly affected the growth performance of the strains both singularly and in combination with other stressing factors since it prolongs the latency phase and reduces the maximum growth rate of strains. Similarly, the oleuropein degradation was mainly affected by the low temperature, especially in presence of low salt content. Despite all strains displayed the ability to reduce the oleuropein content, the beta-glucosidase gene was detected in five out of the nine selected strains, demonstrating that the ability to hydrolyze the oleuropein is not closely related to the presence of beta-glucosidase. Data of the present study suggest that is extremely important to test the technological performances of strains at process conditions in order to achieve a good selection of tailored starter cultures for table olives.

## 1. Introduction

Table olives are the most widespread fermented vegetables in the Mediterranean area and their production and consumption are expanding worldwide, thanks to the nutritional and functional components of drupes, such as polyphenols, vitamins, fiber, minerals, and short chain fatty acids. Olive drupe contains low concentrations of sugar (2.6–6.0%) and high oil (12–30%) and polyphenols content [1,2]. The ladders are mainly represented by oleuropein, which is responsible for the bitterness taste [3] and for inhibiting a range of bacteria, especially lactic acid bacteria (LAB) [4,5,6,7]. Nowadays, depending on the applied industrial process (i.e., Spanish style or Greek style), different debittering methods, enzymatic or chemical, are currently applied [8].

In Sicilian style green table olives, the debittering process is exclusively relied on microorganisms naturally present on the drupes, through the activity of two enzymes, the beta-glucosidase, which leads to the release of two intermediates (glucose and aglycone), which are completely degraded by an esterase into tasteless phenols (hydroxytyrosol and elenolic acid) [9]. Therefore, in order to shorten the debittering step in naturally fermented table olives, tailored-starter cultures, with enhanced debittering ability, are required [10].

It is well known that the use of selected starter cultures, besides to control the fermentation process, should possess the ability to survive in the fermentation environment (low pH, high concentrations of salts, and low fermentation substrates) and to exert acidifying activity (through organic acid production). In addition, they should be able to hydrolyze phenolic compounds (such as oleuropein) and to produce volatile molecules that positively contribute to the development of the sensory profile of the final product [11]. For these reasons, the choice of starter culture tailored for table olive fermentation is a complex assignment, which requires the evaluation of different features since the matrix is very complex and several compounds counter the metabolic activity of the selected strains. Furthermore, the selection of unfitting strains may lead to the production of undesirable metabolites, compromising the final product [12]. Among LAB species, strains belonging to *Lactiplantibacillus plantarum* species are often selected as starters [13,14] for their high versatility, tolerance to stress brine conditions, capacity to cooperate with autochthonous yeasts until the end of the fermentation process, and for their ability to cope with the inhibitory phenolic compounds [15,16,17,18,19,20]. In addition, the presence of genes involved in phenolic-compound degradation has been already demonstrated in some *L. plantarum* strains [21,22,23,24]. This featured is directly correlated with the reduction of the debittering time. In fact, as previously demonstrated by Pino and co-workers (2019), a beta glucosidase-positive strain, ascribed to the *L. plantarum* species and used as starter in low salt fermentation, reduced the processing time and positively affected the polyphenol content and sensory profile of the final product [25].

Up to now, different strains were tested for the ability to degrade the oleuropein under optimal growth conditions [19,26,27] or using MRS medium added with different salt concentrations [16,17,20]. In this context, Yao et al. [28] demonstrated that the tolerance of *L. plantarum* D31 and T9 strains, to 5% and 8% of salt, is related to the presence of specific salt tolerance-related genes.

Recently, Ghabbour and co-workers [29] have evaluated the oleuropein biodegradation ability of the *L. plantarum* FSO175 strain under stress conditions (pH and salt contents), addressing that multi-factor parameters should be taken into account.

In addition to tolerance to pH and salt content, the ability to grow at low temperatures represents another key feature for the selection of starter cultures. Only few studies have been conducted to evaluate the capability of the strains to grow under combined pH and salt conditions at low temperatures [30,31] and, up to now, no data are reported on the impact of technological stress factors on the oleuropein-degrading ability of starter cultures tailored for table olives fermentation.

Therefore, the in order to select tailored starter cultures for table olives fermentation, the objective of the present study was to investigate the effect of olive fermentation-related stress factors (pH, temperature, and NaCl) on growth and on oleuropein-degrading abilities of selected *L. plantarum* strains.

## 2. Materials and Methods

### 2.1. Microorganisms and Culture Conditions

A total of nine *Lactiplantibacillus plantarum* strains, belonging to the Culture Collection of the Department of Agricultural, Food, and Environment (Di3A), University of Catania, Italy were used in this study. The strains derived from a pool of *L. plantarum* isolates from brine samples of naturally fermented Sicilian table olives set up at 5% of NaCl [25], and were selected based on their fermentative abilities on laboratory-scale olives fermentation trials (data not shown). Cultures were maintained as stock solution in 20% (vol/vol) glycerol at –80 °C and routinely propagated at 30 °C for 24 h in De Man, Rogosa and Sharpe (MRS) broth (Oxoid, Milan, Italy).

### 2.2. Growth Ability of L. plantarum Strains under Specific Stress Conditions

The growth ability of the *L. plantarum* strains was tested inoculating each strain at final concentration of 7 log colony forming unit (CFU)/mL in MRS (Oxoid) broth, by using different single and combined stress conditions, such as pH (4.5 and 5.5), salt (NaCl 5% and 6%) and temperatures (16 °C and 32 °C). In detail, MRS broth was acidified adding HCl (0.5 N) and supplemented with 5% or 6% of NaCl, to simulate the salt concentration used during low salt olives fermentation [32]. Growth ability was evaluated after 72 h of incubation at both 16 °C and 32 °C, through the measurement of optical density at 620 nm (iMark™ Microplate Absorbance Reader, Biorad, Milan, Italy) and by plating on MRS agar medium. Each experiment was conducted in triplicate and results were expressed as log CFU/mL ± standard deviation. The condition of pH 6.0 and incubation at 32 °C was used as control, since the specie *L. plantarum* exhibit the best growth optimum at that condition. In order to detect the time required to reach the stationary phase, the *L. plantarum* strains were previously tested in modified MRS medium acidified at pH 6.0 and added with 6% of NaCl, after incubation at 16 °C and 32 °C. The cell density was determined by plating onto MRS agar and growth data ($\mu_{max}$ and λ) were modelled according to the following Gompertz equation:

$$y=k+Aexp\left\{-exp\left[\left(\mu_{max}e/A\right)\left(\lambda -t\right)+1\right]\right\}$$

In the Gompertz equation y is the extent of growth as log CFU/mL at the time t; k is the initial cell density expressed as log CFU/mL; A represents the difference, in cell density, between the stationary phase and the inoculation; $\mu_{max}$ is the maximum growth rate (Δlog CFU/mL/h); *λ* is the length of the latency phase of growth expressed in hours, and *t* is the time.

### 2.3. L. plantarum Beta-Glucosidase Gene Detection

For each *L. plantarum* strain, the presence of the beta-glucosidase gene, encoding for the beta-glucosidase enzyme, was investigated, according to the method proposed by Spano et al. [24]. PCR reactions were carried out in a final volume of 25 µL containing 0.5 U of Taq polymerase, 0.2 mM of dNTPs MIX, 1xPCR buffer, 1.5 mM MgCl_2_, and 0.25 mM of the primer pairs bgluF (5′GTGACTATGGTAGAGTTTCC3′) and bgluR (5′TCAAAACCCATTCCGTTCCCCA3′). The amplification program was as follows: 30 cycles at 94 °C for 1 min, 60 °C for 40 s, and 72 °C 1.2 min, with an initial denaturation at 94 °C for 5 min and a final extension at 72 °C for 10 min. PCR reactions were carried out in a GeneAmp PCR System 2400 (Applied Biosystems, Norwalk, CT, USA). The PCR products were resolved by electrophoresis using 1.2% agarose gel in TAE buffer (0.004 M Tris/acetate, EDTA 1 mM) for about 40 min at 90 V and visualized after staining with Gel Red Nucleic Acid Stain (Biotium, Merck Life Science S.r.l., Milan, Italy). A 200 bp ladder was used as a standard marker.

### 2.4. Oleuropein-Degrading Ability Test

Culture conditions: The degradation of oleuropein was tested by inoculating *L. plantarum* strains (7 log CFU/mL) in modified MRS broth medium, acidified at pH 6.0 and supplemented with 0.1% (*w/v*) (1.0 g/L) of oleuropein, (Sigma, Merck, Life Science S.r.l., Milan, Italy). The MRS medium was modified as follow: 10.0 g/L peptospecial; 10.0 g/L beef extract; 5.0 g/L yeast extract; 20.0 g/L glucose; 2.0 g/L triammonium citrate; 5.0 g/L sodium acetate; 0.2 g/L magnesium sulfate; 0.05 g/L manganese sulfate, and dipotassium phosphate 2.0 g/L (Liofilchem, Roseto degli Abruzzi, Italy). Based on the growth performance of each strain, the assay was carried out at different multi-stress conditions of salt (NaCl 6% and 5%) and temperature (16 °C and 32 °C). After incubation for 72 h, the cultures where centrifuged (8.000 rpm, for 10 min, at 4 °C) and supernatants stored at −20 °C prior to further analyses. Un-inoculated medium was used as control.

HPLC determination of oleuropein: Supernatants were filtered through 0.45 μm PTFE filters (Merck-Millipore, Milan, Italy) and injected into the chromatographic system for HPLC analysis. The HPLC apparatus consisted of a liquid chromatography Water Alliance 2695 HPLC equipped with a Waters 996 photodiode array detector (PDA) set at 280 nm and Waters Empower software. The instrument was provided with a Luna C18 column (250 mm × 4.6 mm i.d., 5 μm, 100 Å, Phenomenex, Torrence, CA, USA) which was maintained at 40 °C in an oven. The flow rate was 1 mL/min. Separation was obtained by elution gradient using an initial composition of 95% of A solution (water acidified with 2% of acetic acid) and 5% of B solution (methanol). The concentration of B solution was increased to 30% in 15 min and to 70% in 25 min and then, after 2 min in isocratic, the mobile phase was set at the initial conditions in 8 min. For quantification in the medium the oleuropein standard (Product Code: 0228 S, Purity for HPLC ≥ 98%) was purchased from Extrasynthese (Genay Cedex, France) [33]. All analyses were performed in triplicate for each sample analyzed and results expressed as mg/L ± standard deviation of oleuropein.

### 2.5. Statistical Analysis

One-way ANOVA followed by Tukey’s multiple comparison test was applied to the data from three biological replicates, using the Statistica software (version 7.0 for Windows, TIBCO Software, Palo Alto, CA, USA) and differences were considered statistically significant at *p* < 0.05.

## 3. Results and Discussion

The increasing demand for healthy food imposes to table olives industry to develop new biotechnological strategies in order to reduce the salt content and to shorten the debittering process, besides reducing chemical treatments. In this contest, the use of *L. plantarum* beta-glucosidase positive strains, as starter cultures, is a promising choice to accelerate the fermentation and to obtain a stable and safety final product [29]. It is well established that *L. plantarum* strains closely fulfill the role of tailored starter culture thanks to its high versatility, adaptation ability, acidic and salt tolerance, and ability to hydrolyze bitter compounds present in olive fruits [15,18,20,34].

One of the main evolutionary strengths of microorganisms is their ability to adapt to changing environments and to tolerate different stress conditions. These abilities are mainly due to the adaptive response of microbial cells through the activation of genes encoding for General Stress Proteins, such as *Dnak, DnaJ, GroES,* and *GroEL* [35,36]. In the present study, nine selected strains belonging to *L. plantarum* specie were investigated for their growth behavior at single and multiple stress conditions, similar to those occurring during olives fermentation, in order to pin point further key factors to be considered for the selection of starters for table olives fermentation.

### 3.1. Growth Performances Exhibited by the Tested Strains

In order to study the growth performance of the tested strains under stress conditions, the Gompertz’s model was applied [37]. Table 1 shows the Gompertz parameters calculated by OD_620_ value collected until 80 h of incubation. However, the growth performances were stopped at 72 h in accordance with the reaching of stationary phase. In detail, when the strain performance (MRS broth medium, pH 6.0) was evaluated at 32 °C and 16 °C, the final average values of cell density were 9.20 and 8.50 log CFU/mL, respectively. According to the final cell density, the A and $\mu_{max}$ values were higher at 32 °C than at 16 °C, whereas the lag phase (λ) was higher at 16 °C than 32 °C, except for the strains F1.16 and F3.8, for which a slightly decrease was observed at the highest tested temperature (32 °C) (Table l).

In addition, in Table 1 growth parameters under combined stress conditions (MRS pH 6.0, added with NaCl 6%, at 32 °C and 16 °C) were reported. Results showed an evident reduction of A and $\mu_{max}$ values, compared to control condition (MRS broth medium, pH 6.0). However, the temperature of 32 °C positively affected the growth performances, in comparison with 16 °C, while λ was higher at 16 °C, except for the strain F3.6, which was negatively affected by 32 °C. The temperature of 16 °C is a key factor for strain’s growth performance since it prolongs the latency phase and reduces the maximum growth rate of strains. By predictive modelling, it was possible to reveal that 72 h is the time required to achieve the maximum exponential phase under stress conditions.

### 3.2. Growth Ability under Stress Conditions

**Figure 1:**
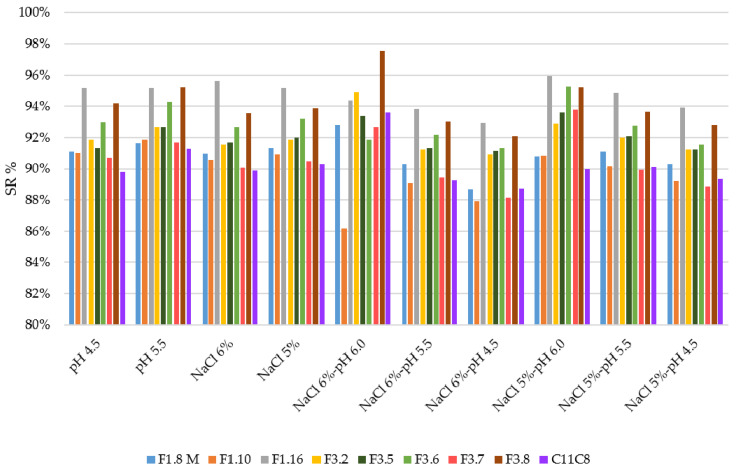
Survival rate plot of the strains, expressed in percentage, under single and combined stress conditions at 32 °C.

**Figure 2:**
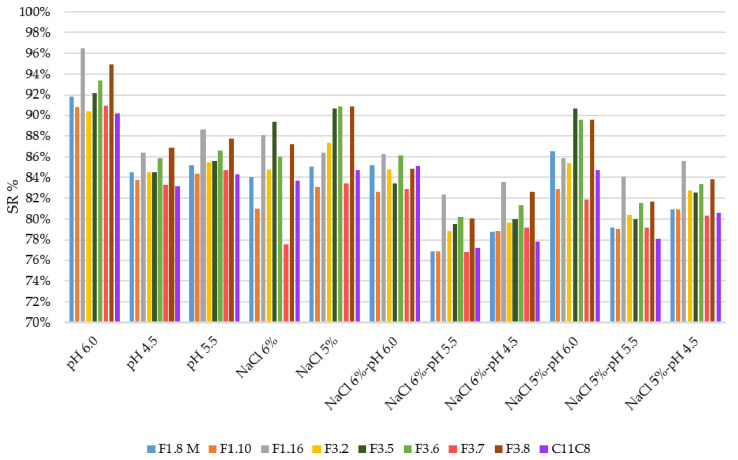
Survival rate plot of the strains, expressed in percentage values, under single and combined stress conditions at 16 °C.

Different studies, conducted on the growth ability of *L. plantarum* species, confirmed the high versatility, adaptation ability, acidic, and salt tolerance as well as ability to decrease the bitter compounds naturally present in olive drupes [15,16,17,18,20]. In the present study, nine (9) selected *L. plantarum* strains were investigated for their growth behavior at single and combined stress conditions simulating the table olives fermentation. The growth ability of the tested strains at 32 °C and 16 °C, as reported above, was monitored up to 72 h and results are displayed in Supplementary Figures S1 and S2. At control condition (pH 6.0 and incubation at 32 °C) all tested strains, starting from an average value of 7 log CFU/mL, increased their cell density more than 2 log units. Overall, all strains appeared more resistant toward both single and multi-stress conditions at 32 °C (Figure S1), showing a mean increase of cell densities higher than 1 log unit. In order to evaluate the growth ability of each tested strains under both single and multi-stress conditions, the survival rate percentage (SR %) was calculated based on viable cells under control condition (pH 6.0 and incubation at 32 °C) and under each stressor condition. The viable cells were enumerated by plate count on MRS agar. Figure 1 and Table S1 show the SR % and the viable count (log CFU/mL) of the tested strains under each stressor at 32 °C, respectively. Overall, all strains showed the ability to survive under the tested stress conditions with SR % more than 85%. Zooming on each stress factor, out of the nine strains, 2 (F1.16 and F3.8) showed the highest SR % under all the tested stress conditions. Similar behavior was exhibited by the F3.2 strain except at NaCl 6% and pH 6.0. The performances of the tested strains appeared to be robust toward the different stressors, in accordance with previous studies, which validated the aptitude of *L. plantarum* species to tolerate acidic environments, for the intrinsic ability to maintain an internal pH gradient that allows the survival at a much lower external pH [26]. A recent study [38], carried out on *L. plantarum* KLOS 1.0328 strain, showed its greater inclination to tolerate acidic stress rather than osmotic stress, according to a previous work reporting that pH values from 5.0 to 9.0 did not significantly affect the growth of *L. plantarum* strains isolated from Italian Bella di Cerignola table olives [30]. However, the same authors asserted that salt concentrations higher than 4% negatively influenced the strain growth. On the contrary, other studies [39,40,41] reported the ability of *L. plantarum* strains to tolerate high (> 8%) NaCl concentrations. The tolerance to high salt concentrations and the ability to control osmotic stress have been recently confirmed [28], showing excellent growth performance of *L. plantarum* strains at 5% and 8% of NaCl. The authors observed that *L. plantarum* strains are equipped, at genomic level, with a complex molecular regulatory network involving genes associated with salt resistance through the recovery of the intracellular ion balance [28]. Our study clearly highlights that the growth behavior under stress conditions is strain-specific, especially at 16 °C (Figure 2 and Table S2). By including the temperature of 16 °C, as an additional stressor, data revealed that the growth performance detected among strains, was highly variable, appearing to be hampered by the multi-stress conditions. Moreover, comparing the behavior of each strain at different stress conditions, the growth performance, expressed as viable counts, was highly variable. Based on ANOVA results the strains F3.8, F3.7, and F1.16 showed the higher significant differences (Table S2). As expected, when incubated at 16 °C, the viable count at pH 6.0 was significantly lower than those observed at 32 °C (Table S2). Indeed, the low temperature significantly affected the growth performance of the strains both singularly and in combination with other stressing factors. Moreover, it is interesting to point out that at 16 °C under acidic conditions the growth of the *L. plantarum* strains was significantly affected. Our data were slightly in discordance with previous reports [30,31] that revealed high growth ability of *L. plantarum* strains at both 12 °C and 15 °C. Although both table olives process conditions and storage temperature may vary in relation to annual climate fluctuations, to type of containers and to industrial sheds, the most common temperature conditions measured during the fermentation process and storage period is about 20 °C. Hence, it is extremely important to select *L. plantarum* strains with enhanced technological performances at process parameters applied during table olives process production.

### 3.3. Molecular Detection of Beta-Glucosidase Gene in L. plantarum Strains

According to the PCR protocol proposed by Spano et al. [24], for each *L. plantarum* strains the presence of the beta-glucosidase gene was investigated. The gene, encoding for a beta-glucosidase enzyme, has been selected for the relevance of oleuropeinolytic activity, towards phenolic glucosides in table olives, such as oleuropein, demethyl-oleuropein, verbascoside, and luteolin-7-glucosides [21,26,42,43]. PCR reactions were performed with either degenerated primers, deduced from the nucleotide sequences of beta-glucosidase genes identified for *L. plantarum* species. A single PCR-product (of about 1400 bp) was obtained using the primer pairs designed on the putative beta-glucosidase gene of the *L. plantarum* WCFS1 strain. High identity value was observed between nucleotide sequences of *L. plantarum* tested strains.

The beta-glucosidase gene is a ubiquitous gene detected on strains isolated from both vegetable and dairy products [26]. Among the nine strains tested for the beta-glucosidase activity, five strains (F1.8M, F3.2, F3.5, F3.8, and C11C8) demonstrated the presence of the gene encoding for the beta-glucosidase activity. However, for four tested strains (F1.10, F1.16, F3.6, and F3.7) the beta-glucosidase gene was not detected, despite their displayed the ability to reduce the oleuropein content, under stress conditions. Similar results were obtained by Carrasco and co-workers [44] who demonstrated that the *Lactiplantibacillus pentosus* CECT4023 strain, although in absence of the gene encoding for the beta-glucosidase activity, is able to metabolize the oleuropein. This observation can be explained taking into account that the ability to hydrolyze the oleuropein is not closely related to the presence of beta-glucosidase and could be led to the activity of tannases and esterases enzymes [20,45,46].

### 3.4. Oleuropein Degrading Test

In selection process of tailored-starter cultures for table olives fermentation, the ability to degrade the oleuropein, present in drupes, is one of the key characteristic to be considered in order to shorten the debittering stage. In the present work, based on the growth performances, nine *L. plantarum* strains were subjected to the oleuropein degrading test at the following combined conditions: (1) pH 6.0 and NaCl 6%, (2) pH 6.0 and NaCl 5%, and both incubated at 32 °C and 16 °C. In Table 2 shows data on detected concentrations of oleuropein (OLE), expressed as mg/L, and on the OLE degradation, expressed as percentage. Overall, different degradation ability was detected among the tested strains, indicating a strain-specific behavior. In particular, at 32 °C, in presence of both 5% and 6% of NaCl, all strains showed the ability to degrade the oleuropein albeit the highest OLE degradation percentages were detected in presence of 6% of NaCl. In detail, as reported in Table 2, the strain F1.16 exhibited the highest OLE degradation percentage (97.8%). Zooming on the results obtained at 16 °C, low OLE degrading activity was revealed for almost all the tested strains at both 5% and 6% of NaCl with the exception of the F3.2 strain. In detail, at 5.0% of NaCl and at pH 6.0, the F1.10 strain, maintained the highest OLE degradation activity (88.8%), followed by F1.16, F3.2, and C11C8 strains, whereas the F1.8M, F3.7, and F3.8 strains showed lower OLE degradation performances, with degradation percentage values of 83.2%, 78.3%, and 84.7%, respectively. The lowest tested temperature (16 °C) negatively affected the OLE degrading ability exhibited by the F3.5 and F3.6 strains, with percentage values of 18.7% and 20.3%, respectively.

The OLE degrading ability of *L. plantarum* strains, has been already confirmed at 30 °C by several authors [19,26] and only recently Ghabbour et al. [29] investigated the performance of *L. plantarum* strains under combined stress factors (such as pH and salt concentrations), revealing a good degrading ability at pH 4.5 and in presence of 5% of NaCl. However, Iorizzo et al. [27] showed that the use of nutrient medium at pH 5.0, supplemented with oleuropein, shorten the OLE degradation time. Our results indicated a high degrading ability of the tested strains on modified MRS medium supplemented with oleuropein at pH 6.0. In addition, the salt content could improve the ability of the strains to degrade the oleuropein, when multi-stress conditions are occurring.

Up to now, there is no scientific evidence on the ability of *L. plantarum* strains to degrade oleuropein at low temperature. The present study clearly revealed that the temperature is a key parameter, which could be proposed for the selection of tailored starter cultures for table olives fermentation.

## 4. Conclusions

In the present study, nine selected *L. plantarum* strains were evaluated for the ability to growth and to degrade the oleuropein under stress conditions. Our data demonstrated that the behavior of the selected strains was strain-dependent for all the tested stressors. The low temperature was the main stress factor affecting the survival rate, under simulated brine conditions. Regarding to the oleuropein degradation ability, it is interesting to highlight that out of the nine strains, 3 (F1.10, F1.16, and F3.7) showed high percentage of degraded OLE, even the beta-glucosidase gene was not detected. Therefore, further proteomics and genomics studies are ongoing to reveal gene loci related to oleuropein degradation.

Based on the challenging brine environment factors, considered in the present study, the F1.16 and F3.8 strains are promising candidate as tailored starter culture for table olives.

## Figures and Tables

**Table 1:** Growth Parameters, Calculated by Gompertz’s Equation of *L. plantarum* Strains under Control Conditions (MRS Broth Medium pH 6.0) and under Stress Condition (MRS Broth Medium pH 6.0 and NaCl 6%) at 32 °C and 16 °C.

*L. plantarum* Strains	Control Condition (MRS Broth Medium pH 6.0)						Stress Condition (MRS Broth Medium pH 6.0 and NaCl 6%)					
	*A*		*μ_max_*		*λ*		*A*		*μ_max_*		*λ*	
	32 °C	16 °C	32 °C	16 °C	32 °C	16 °C	32 °C	16 °C	32 °C	16 °C	32 °C	16 °C
**F1.8M**	2.63 ± 0.02	0.43 ± 0.02	0.16 ± 0.02	0.01 ± 0.01	13.57 ± 0.08	20.02 ± 0.02	0.51 ± 0.04	0.46 ± 0.02	0.03 ± 0.02	0.03 ± 0.02	29.99 ± 0.07	32.91 ± 0.07
**F1.10**	2.79 ± 0.02	0.38 ± 0.03	0.15 ± 0.03	0.02 ± 0.02	13.20 ± 0.05	20.80 ± 0.15	0.14 ± 0.02	0.12 ± 0.03	0.03 ± 0.02	0.03 ± 0.03	22.82 ± 0.06	24.15 ± 0.04
**F1.16**	1.15 ± 0.03	0.45 ± 0.02	0.05 ± 0.02	0.02 ± 0.01	21.42 ± 0.04	19.96 ± 0.02	0.32 ± 0.03	0.29 ± 0.02	0.02 ± 0.02	0.02 ± 0.02	20.92 ± 0.06	22.77 ± 0.03
**F3.2**	2.26 ± 0.02	0.26 ± 0.02	0.09 ± 0.02	0.02 ± 0.02	16.06 ± 0.03	20.23 ± 0.03	0.75 ± 0.03	0.59 ± 0.02	0.02 ± 0.01	0.01 ± 0.01	23.73 ± 0.05	26.22 ± 0.04
**F3.5**	2.25 ± 0.02	0.50 ± 0.02	0.09 ± 0.02	0.01 ± 0.01	16.07 ± 0.02	18.76 ± 0.02	0.51 ± 0.03	0.49 ± 0.02	0.03 ± 0.03	0.07 ± 0.02	30.03 ± 0.03	31.48 ± 0.08
**F3.6**	1.47 ± 0.05	0.38 ± 0.05	0.05 ± 0.04	0.02 ± 0.02	19.11 ± 0.02	24.29 ± 0.04	0.28 ± 0.02	0.25 ± 0.02	0.01 ± 0.01	0.01 ± 0.01	30.03 ± 0.05	24.16 ± 0.05
**F3.7**	2.87 ± 0.02	0.44 ± 0.04	0.18 ± 0.02	0.08 ± 0.07	14.12 ± 0.03	15.50 ± 0.05	0.58 ± 0.02	0.49 ± 0.03	0.02 ± 0.01	0.02 ± 0.02	23.80 ± 0.06	25.29 ± 0.06
**F3.8**	1.17 ± 0.04	0.43 ± 0.03	0.05 ± 0.04	0.02 ± 0.01	21.29 ± 0.03	19.83 ± 0.03	0.74 ± 0.04	0.52 ± 0.04	0.02 ± 0.02	0.02 ± 0.02	22.98 ± 0.11	24.47 ± 0.07
**C11C8**	2.39 ± 0.03	0.38 ± 0.02	0.16 ± 0.04	0.02 ± 0.02	13.35 ± 0.04	24.26 ± 0.06	0.80 ± 0.04	0.60 ± 0.06	0.03 ± 0.01	0.02 ± 0.01	21.22 ± 0.03	22.89 ± 0.06

**Table 2:** Detected Values of Oleuropein (OLE) (Expressed as mg/L) and Percentage of Degraded OLE from Strains Inoculated in Modified MRS Medium and Incubated at Different Temperatures (32 °C and 16 °C).

	MRS Broth, 0.1% (*w/v*) of OLE, NaCl 5.0% (*w/v*), pH 6.0				MRS Broth, 0.1% (*w/v*) of OLE, NaCl 6.0% (*w/v*), pH 6.0			
	32 °C		16 °C		32 °C		16 °C	
	OLE (mg/L)	OLE (%)	OLE (mg/L)	OLE (%)	OLE (mg/L)	OLE (%)	OLE (mg/L)	OLE (%)
**Control**	975.0 ± 2.71 ^a^	00.0	985.0 ± 3.00 ^a^	00.0	978.0 ± 2.71 ^a^	00.0	983.0 ± 3.00 ^a^	00.0
***L. plantarum* Strains**								
F1.8M	39.8 ± 0.04 ^h^	95.9	165.7 ± 11.98 ^d^	83.2	44.9 ± 2.83 ^g,h^	95.4	93.7 ± 6.47 ^d^	90.5
F1.10	45.6 ± 1.60 ^h^	95.3	110.3 ± 13.60 ^f,g^	88.8	66.9 ± 2.56 ^e,f^	93.2	93.9 ± 13.31 ^d^	90.4
F1.16	82.7 ± 0.25 ^g^	91.5	123.7 ± 1.05 ^e,f^	87.4	21.7 ± 0.51 ^i^	97.8	38.8 ± 0.91 ^g,h,i^	96.0
F3.2	129.8 ± 0.64 ^e,f^	86.7	125.9 ± 0.55 ^e,f^	87.2	46.8 ± 1.19 ^g^	95.2	49.6 ± 1.57 ^f,g^	95.0
F3.5	102.3 ± 0.18 ^f,g^	89.5	801.2 ± 10.52 ^b^	18.7	46.8 ± 0.77 ^g^	95.2	752.8 ± 2.54 ^b^	23.4
F3.6	84.8 ± 2.66 ^g^	91.3	785.0 ± 7.72 ^b^	20.3	26.9 ± 1.57 ^i^	97.3	723.0 ± 3.28 ^c^	26.4
F3.7	41.2 ± 3.57 ^h^	95.8	213.4 ± 21.63 ^c^	78.3	69.1 ± 0.26 ^e^	92.9	94.7 ± 0.11 ^d^	90.4
F3.8	44.8 ± 1.85 ^h^	95.4	150.8 ± 1.51 ^d,e^	84.7	35.5 ± 4.11 ^g,h,i^	96.4	96.8 ± 8.34 ^d^	90.2
C11C8	88.1 ± 0.13 ^g^	91.0	126.6 ± 2.26 ^e,f^	87.1	27.9 ± 0.61 ^h,i^	97.2	49.3 ± 1.37 ^g^	95.2

a–i: different letters within the same column indicate significant differences at *p* < 0.05.

## Supplementary Materials

The following are available online at https://www.mdpi.com/2076-2607/8/10/1607/s1, Figure S1: Box plot of cell density (expressed as log CFU/mL) of *L. plantarum* strains, at different pH and salt conditions at 32 °C, Figure S2: Box plot of cell density (expressed as log CFU/mL) of *L. plantarum* strains, at different pH and salt conditions at 16 °C, Table S1: Viable counts (log CFU/mL) of *L. plantarum* strains at different stress conditions, incubated at 32 °C. All strains, as reported in the text, were at initial cell density of 7 log CFU/mL, Table S2: Viable counts (log CFU/mL) of *L. plantarum* strains at different stress conditions, incubated at 16 °C. All strains, as reported in the text, were at initial cell density of 7 log CFU/mL.
