# Supplementary material for: Effects of Different Stress Parameters on Growth and on Oleuropein-Degrading Abilities of Lactiplantibacillus plantarum Strains Selected as Tailored Starter Cultures for Naturally Table Olives
Source: Microorganisms. 2020 Oct 19;8(10):1607. doi: 10.3390/microorganisms8101607 (PMC7590217; doi:10.3390/microorganisms8101607)
Supplement: Supplementary file 1 [file microorganisms-08-01607-s001.pdf]

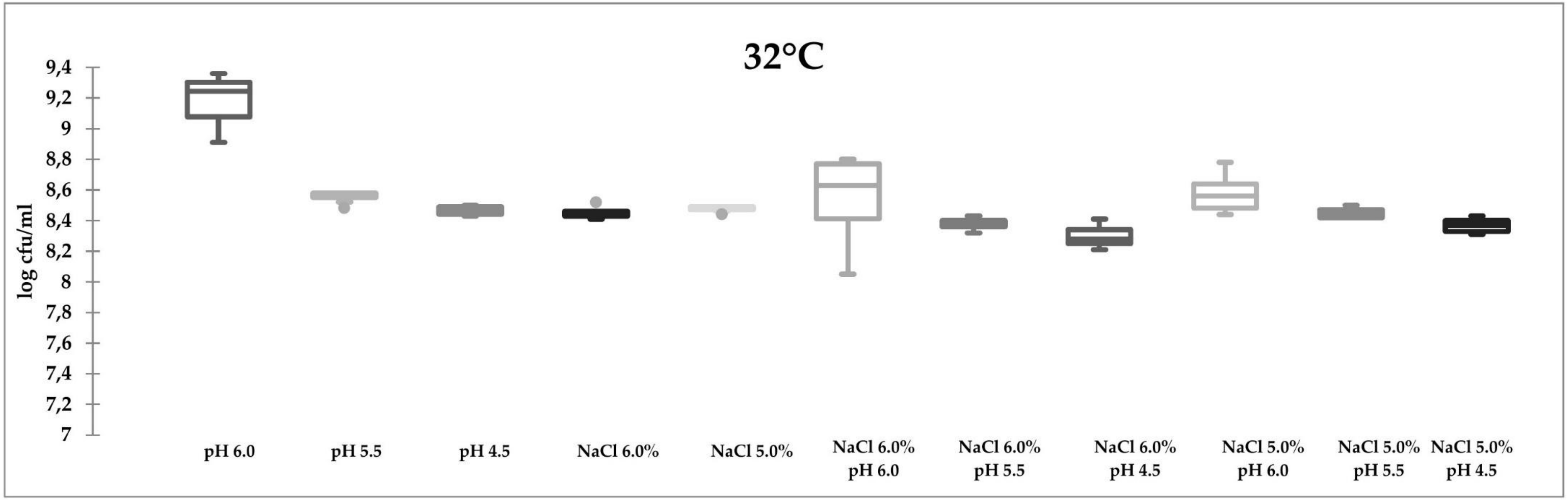

Figure S1: Box plot of cell density (expressed as log CFU/mL) of *L. plantarum* strains, at different pH and salt conditions at 32 °C.

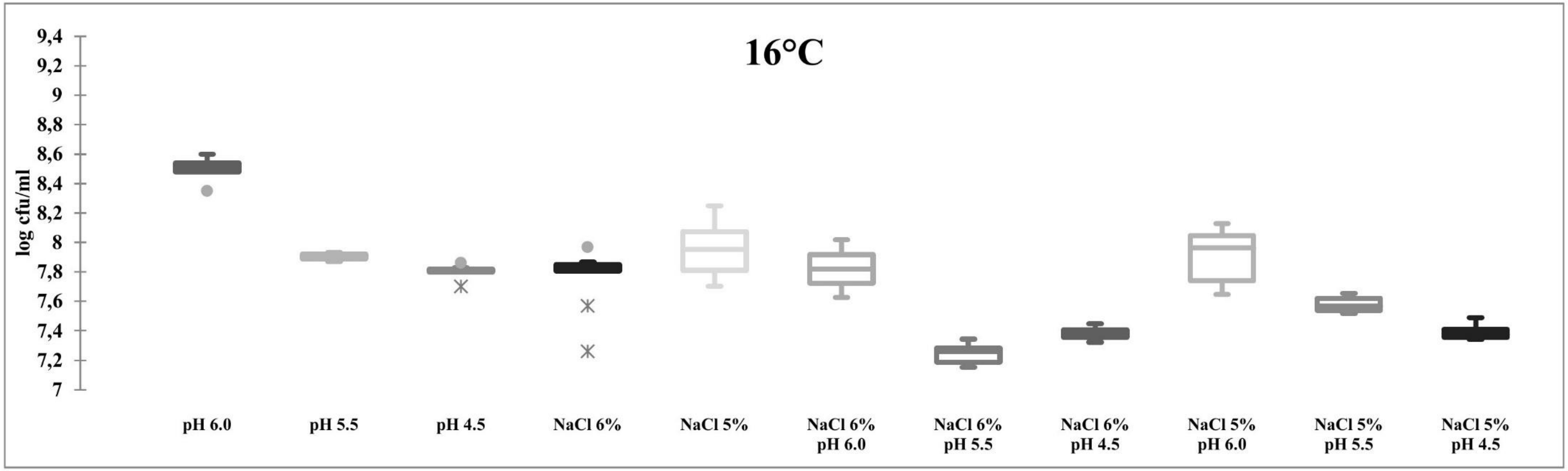

Figure S2: Box plot of cell density (expressed as log CFU/mL) of *L. plantarum* strains, at different pH and salt conditions at 16 °C

Supplementary Table 1. Viable counts (log CFU/mL) of *L. plantarum* strains at different stress conditions, incubated at 32°C. All strains, as reported in the text, were inoculated at initial cell density of 7 log CFU/mL.

***L. plantarum* strains**						NaCl 6.0%			NaCl 5.0%		
	pH 6.0	pH 5.5	pH 4.5	NaCl 6.0%	NaCl 5.0%	pH 6.0	pH 5.5	pH 4.5	pH 6.0	pH 5.5	pH 4.5
F1.8M	9.30±0.07^{aAB}	8.52±0.10^{bc}	8.47±0.06^{bc}	8.46±0.05^{bc}	8.49±0.06^{bc}	8.63±0.19^{bAB}	8.40±0.07^{bc}	8.25±0.07^{c}	8.44±0.13^{bc}	8.47±0.05^{bc}	8.40±0.06^{bc}
F1.10	9.34±0.12^{aA}	8.58±0.06^{b}	8.50±0.10^{b}	8.46±0.07^{bc}	8.49±0.06^{b}	8.05±0.11^{dC}	8.32±0.09^{bcd}	8.21±0.12^{cd}	8.48±0.11^{bc}	8.45±0.06^{bc}	8.33±0.11^{bc}
F1.16	8.91±0.06^{aD}	8.48±0.07^{ab}	8.48±0.07^{ab}	8.52±0.09^{ab}	8.48±0.06^{ab}	8.41±0.12^{bABC}	8.36±0.06^{b}	8.28±0.14^{b}	8.55±0.20^{ab}	8.45±0.06^{ab}	8.37±0.06^{b}
F3.2	9.24±0.06^{aAB}	8.56±0.05^{bc}	8.49±0.08^{c}	8.46±0.07^{c}	8.49±0.05^{c}	8.77±0.19^{bAB}	8.43±0.06^{c}	8.40±0.07^{c}	8.58±0.09^{bc}	8.50±0.05^{c}	8.43±0.07^{c}
F3.5	9.23±0.13^{aABC}	8.55±0.07^{b}	8.43±0.08^{b}	8.46±0.06^{b}	8.49±0.05^{b}	8.62±0.26^{bAB}	8.43±0.12^{b}	8.41±0.06^{b}	8.64±0.20^{b}	8.50±0.05^{b}	8.42±0.13^{b}
F3.6	9.08±0.06^{aBCD}	8.56±0.06^{bc}	8.44±0.07^{bcd}	8.41±0.07^{bcd}	8.46±0.08^{bcd}	8.34±0.11^{cBC}	8.37±0.05^{cd}	8.29±0.07^{d}	8.65±0.14^{b}	8.42±0.06^{bcd}	8.31±0.09^{d}
F3.7	9.36±0.10^{aA}	8.58±0.06^{bcd}	8.49±0.09^{cde}	8.43±0.07^{cde}	8.47±0.07^{cde}	8.67±0.12^{bcAB}	8.37±0.08^{de}	8.25±0.1^{e}	8.78±0.11^{b}	8.42±0.06^{cde}	8.32±0.09^{de}
F3.8	8.99±0.08^{aCD}	8.56±0.06^{bc}	8.47±0.07^{cd}	8.41±0.06^{cd}	8.44±0.08^{cd}	8.77±0.15^{abAB}	8.36±0.07^{cd}	8.28±0.07^{d}	8.56±0.16^{bc}	8.42±0.06^{cd}	8.34±0.09^{cd}
C11C8	9.30±0.10^{aAB}	8.58±0.07^{bc}	8.44±0.09^{c}	8.45±0.06^{b}	8.49±0.05^{b}	8.80±0.05^{bA}	8.39±0.12^{b}	8.34±0.06^{b}	8.46±0.15^{b}	8.47±0.06^{b}	8.40±0.10^{b}

a-e: different lowercase letters within the same line indicate significant differences at $P < 0.05$; A-D: different capital letters within the same column indicate significant differences at $P < 0.05$.

Supplementary Table 2. Viable counts (log CFU/mL) of *L. plantarum* strains at different stress conditions, incubated at 16°C. All strains, as reported in the text, were inoculated at initial cell density of 7 log CFU/mL.

L. plantarum strains						NaCl 6.0%			NaCl 5.0%		
	pH 6.0	pH 5.5	pH 4.5	NaCl 6.0%	NaCl 5.0%	pH 6.0	pH 5.5	pH 4.5	pH 6.0	pH 5.5	pH 4.5
F1.8M	8.54±0.12^{a}	7.92±0.05^{b}	7.86±0.06^{b}	7.81±0.12^{bB}	7.91±0.07^{bBCD}	7.92±0.07^{bAB}	7.15±0.08^{d}	7.32±0.09^{cd}	8.05±0.10^{bBC}	7.53±0.05^{c}	7.36±0.06^{cd}
F1.10	8.48±0.06^{a}	7.88±0.05^{b}	7.83±0.06^{b}	7.57±0.10^{cdBC}	7.76±0.07^{bcD}	7.72±0.12^{bcBC}	7.18±0.1^{e}	7.36±0.08^{de}	7.74±0.05^{bcDE}	7.56±0.05^{dc}	7.38±0.07^{de}
F1.16	8.60±0.05^{a}	7.90±0.05^{b}	7.70±0.05^{cd}	7.85±0.06^{bcB}	7.70±0.05^{cdD}	7.69±0.15^{cdBC}	7.34±0.09^{f}	7.45±0.06^{f}	7.65±0.10^{edE}	7.63±0.06^{ef}	7.49±0.06^{ef}
F3.2	8.35±0.20^{a}	7.90±0.05^{bc}	7.81±0.06^{bc}	7.83±0.11^{bcB}	8.07±0.09^{bABCD}	7.83±0.06^{bcABC}	7.28±0.07^{e}	7.36±0.09^{e}	7.89±0.05^{bcCD}	7.65±0.05^{dc}	7.43±0.09^{de}
F3.5	8.51±0.11^{a}	7.90±0.05^{b}	7.80±0.06^{bc}	8.25±0.07^{aA}	8.37±0.07^{aA}	7.70±0.14^{bcBC}	7.34±0.09^{d}	7.38±0.07^{d}	8.37±0.09^{aA}	7.62±0.05^{c}	7.38±0.07^{d}
F3.6	8.48±0.06^{a}	7.87±0.06^{c}	7.80±0.05^{c}	7.81±0.06^{cB}	8.25±0.05^{bAB}	7.82±0.07^{cABC}	7.28±0.08^{e}	7.38±0.07^{e}	8.13±0.06^{bB}	7.57±0.06^{d}	7.40±0.07^{de}
F3.7	8.51±0.14^{a}	7.93±0.04^{b}	7.8±0.07^{bc}	7.26±0.12^{fC}	7.81±0.07^{bcCD}	7.76±0.07^{bcBC}	7.19±0.10^{f}	7.41±0.06^{ef}	7.66±0.11^{cdE}	7.52±0.05^{de}	7.41±0.08^{ef}
F3.8	8.54±0.13^{a}	7.89±0.06^{c}	7.81±0.07^{cd}	7.84±0.10^{dcB}	8.17±0.06^{bABC}	7.63±0.10^{deC}	7.20±0.05^{g}	7.43±0.06^{fg}	8.05±0.11^{bcBC}	7.54±0.06^{ef}	7.34±0.07^{fg}
C11C8	8.48±012^{a}	7.92±0.06^{b}	7.82±0.08^{b}	7.87±0.06^{bB}	7.96±0.07^{bBCD}	8.00±0.10^{bA}	7.26±0.09^{d}	7.32±0.07^{d}	7.96±0.05^{bBC}	7.58±0.05^{c}	7.34±0.11^{d}

a-g: different lowercase letters within the same line indicate significant differences at $P < 0.05$**;** A-E different capital letters within the same column indicate significant differences at $P < 0.05$.
